# Failure to thrive - an overlooked manifestation of *KMT2B*-related dystonia: a case presentation

**DOI:** 10.1186/s12883-020-01798-x

**Published:** 2020-06-16

**Authors:** Andrew Ng, Serena Galosi, Lisa Salz, Terence Wong, Caitlin Schwager, Shivarajan Amudhavalli, Rose Gelineau-Morel, Shimul Chowdhury, on behalf of Rady Children’s Institute for Genomic Medicine Investigators, Jennifer Friedman

**Affiliations:** 1 University of California San Diego, San Diego, CA USA; 2 Rady Children’s Hospital, San Diego, CA USA; 3 Sapienza University, Rome, Italy; 4 Rady Children’s Institute for Genomic Medicine, San Diego, CA USA; 5 Children’s Mercy Hospital, Kansas City, MO USA

**Keywords:** KMT2B, Dystonia, Failure to thrive, Whole genome sequencing

## Abstract

**Background:**

*KMT2B*-related dystonia is a recently described form of childhood onset dystonia that may improve with deep brain stimulation. Prior reports have focused on neurologic features including prominent bulbar involvement without detailing general health consequences that may result from orolingual dysfunction. We describe a family with novel *KMT2B* mutation with several members with failure to thrive to highlight this non-neurologic, but consequential impact of mutation in this gene.

**Case presentation:**

We present a case of a 15-year old female who was admitted and evaluated for failure to thrive. On exam, she had severe speech dysfluency, limited ability to protrude the tongue, and generalized dystonia involving the oromandibular region, right upper and left lower extremity with left foot inversion contracture. The proband and her parents underwent whole genome sequencing. A previously undescribed variant, c.4960 T > C (p.Cys1654Arg), was identified in the *KMT2B* gene in the proband and mother, and this variant was subsequently confirmed in two maternal cousins, one with failure to thrive. Literature review identified frequent reports of prominent bulbar involvement but failure to thrive is rarely mentioned.

**Conclusion:**

Failure to thrive is a common pediatric clinical condition that has consequences for growth and development. In the presence of an abnormal neurologic exam, a search for a specific underlying genetic etiology should be pursued. With this case series, we highlight an unusual potentially treatable cause of failure to thrive, reinforce the importance of precise molecular diagnosis for patients with failure to thrive and an abnormal neurologic exam, and underscore the importance of cascade screening of family members.

## Background

Failure to thrive (FTT), a common clinical condition warrants hospitalization to ensure adequate nutrition and thorough investigation of etiology. FTT is defined as weight less than 0.4-5th percentile, weight less than 80% normal weight for age, or weight decline across more than 2 major percentiles [1]. The causes include poor nutrition, inadequate absorption, and increased energy expenditure. Careful attention to history and comprehensive physical exam can yield clues to etiology.

Dystonia is a movement disorder characterized by involuntary hyperkinetic movements involving sustained or intermittent contractions of agonist and antagonist muscles that frequently lead to abnormal posturing or movements [2]. Dystonia is classified based on clinical characteristics (age of onset, regional distribution, temporal pattern, coexistence of other movement disorders, and other neurological manifestations) and etiology (genetic, acquired, or idiopathic) [3]. Orolingual dystonia can cause eating dysfunction leading to weight loss [4].

Lysine Methyltransferase-2B (*KMT2B)* dystonia is a recently described autosomal dominant disorder [5, 6]. The *KMT2B* gene encodes a lysine methyltransferase involved in H3K4 methylation, an epigenetic modifier active in development [7]. This condition is characterized by childhood lower-limb onset dystonia that progressively generalizes with prominent cranial, cervical, and laryngeal involvement [5, 6]. Though dysphagia has been described, reports have focused on neurologic rather than gastrointestinal symptomatology and presentation. We report the first case series where failure to thrive was the presenting feature prompting diagnosis. Our report identifies a novel *KMT2B* pathogenic variant, c.4960 T > C (p.Cys1654Arg), and expands both the spectrum of phenotypic presentation for *KMT2B* mutation and genetic causes for FTT.

## Case presentation

A 15-year-old girl (III-1) was admitted to the hospital for FTT: 36 kg(0.18%ile), 151 cm (4%ile). She had not seen a physician for 2 years due to socioeconomic issues. History revealed mild cognitive impairment, gait abnormality (left foot inversion) onset age 3, speech dysfluency onset age 9, and slowness with eating onset age 13 with dysphagia to solids and liquids onset age 14. Previous evaluation for gait abnormality resulted in unsuccessful trials of muscle relaxants and orthotics. Family history was initially negative for neurologic conditions. On exam, there was severe speech dysfluency, limited ability to protrude the tongue, and generalized dystonia involving the oromandibular region, the right upper and the left lower extremity with left foot inversion contracture (Additional files 1, 2, 3).


**Additional file 1.** Patient (III-1) demonstrates orolingual dysphonia and dystonia. In the last segment, she is asked to protrude her tongue but cannot.



**Additional file 2.** Patient (III-1) demonstrates upper extremity dystonia of right arm and lower extremity of left leg  while sitting.



**Additional file 3.** Patient (III-1) demonstrates left lower limb dystonia while ambulating.


**Fig. 1:**
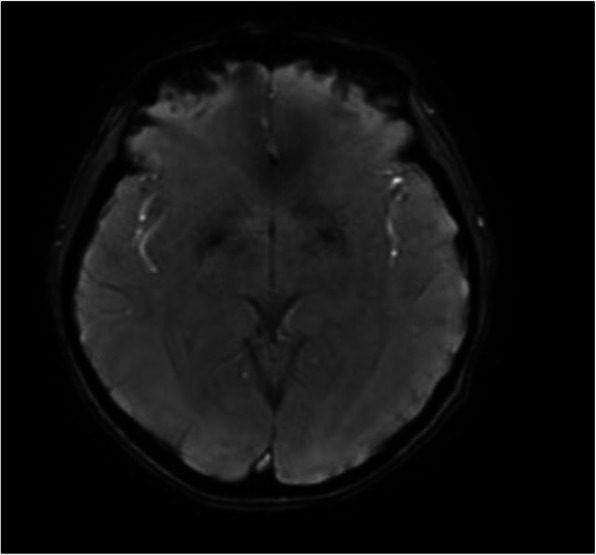
Proband (III-1) MRI brain susceptibility weighted imaging demonstrates moderate symmetric hypointensity in the bilateral globi pallidi

Initially, neglect was considered a possible etiology given the delay in seeking medical evaluation. However, abnormal neurologic exam prompted further testing. Labs were normal except for mild thrombocytopenia likely due to malnutrition (Table 1). Brain magnetic resonance imaging (MRI) showed bilateral hypointensity in globi pallidi on susceptibility-weighted imaging supporting an organic etiology (Fig. 1). Radiofilm revealed left foot 5th metatarsal fracture. Muscle biopsy showed myopathic fiber size variation and mild vasculopathic changes. Video-swallow fluoroscopy showed dysphagia to liquids and solids. Gastrostomy tube was placed with significant weight gain but persistence of weight below the second percentile despite appropriate caloric intake. Levodopa/carbidopa and trihexyphenidyl were not beneficial. Trio whole genome sequencing (WGS) revealed a novel likely pathogenic heterozygous c.4960 T > C (p.Cys1654Arg) variant in the proband and mother in the *KMT2B* gene (Transcript ID: NM_014727.2*)*. This variant is not present in the gnomAD database. The c.4960 T > C (p.Cys1654Arg) variant was predicted by multiple in silico tools to have a deleterious effect on protein function. No other diagnostic variants were identified.

**Table 1:** Laboratory Values during hospital admission

LABORATORY TEST	VALUE	REFERENCE RANGE
Hemoglobin (g/dL)	14.5	12.5–15
Hematocrit (% of g/dL)	42.2	35–45
Platelets (K/uL)	135	140–440
Sodium (mmol/L)	141	133–143
Potassium (mmol/L)	4.1	3.4–4.7
Chloride (mmol/L)	105	98–106
BUN (mg/dL)	9	8–21
Creatinine (mg/dL)	0.65	0.6–1.2
Calcium (mg/dL)	9.5	8.5–10.4
AST (U/L)	26	15–30
ALT (U/L)	20	5–30
Alkaline phosphatase (U/L)	99	70–280
Glucose (mg/dL)	93	70–106
Albumin (g/dL)	4.7	3.5–5.1
Vitamin D 25 OH (ng/mL)	17	> 30
TSH (uIU/mL)	0.37	0.35–5
Free T4 (ng/dL)	0.75	0.71–1.85
CK (U/L)	44	20–128
Folate (ng/mL)	11.7	> 8
Vitamin B12 (pg/mL)	480	260–935
Copper (mcg/dL)	87	75–187
Ceruloplasmin (mg/dL)	21	21–46

*AST* aspartate transaminase, *ALT* alanine aminotransferase; *Vitamin D 25 OH* calcifediol, *TSH* thyroid stimulating hormone, *CK* creatinine kinase

**Fig. 2:**
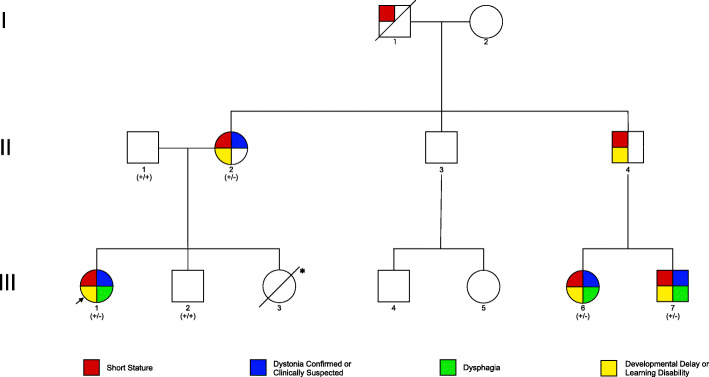
Family Pedigree; Core features of *KMT2B* related dystonia are highlighted. + wild type; − *KMT2B* c.4960 T > C (p.Cys1654Arg) variant carrier; * full term stillbirth. Individuals II-2; III-1, III-2; III-6; III-7 were directly examined. Features reported in other individuals are by report of relatives

After identification of the *KMT2B* variant in the proband and mother, additional history was obtained. A three-generation pedigree was constructed (Fig. 2). Cascade testing of maternal cousins (III-6 and III-7) revealed that they carried the same *KMT2B* c.4960 T > C (p.Cys1654Arg) variant. The father (II-4) of maternal cousins (III-6 and III-7) is an obligate carrier.

**Fig. 3:**
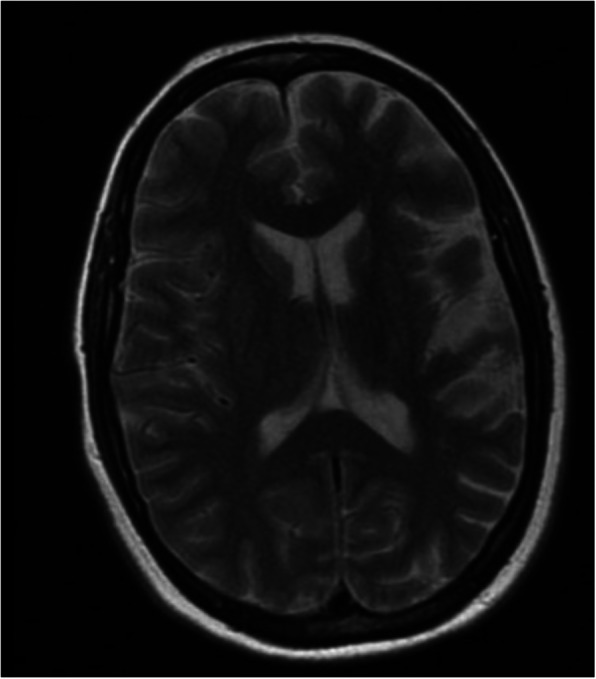
Mother (II-2) MRI brain showing T2 hyperintensity without enhancement in the deep and subcortical white matter of the left frontal lobe suggesting remote infarct in the left middle cerebral territory


Mother, age 34(II-2), relayed history of painful right arm posturing, worsening handwriting, intermittent numbness, and gait disturbance onset age 29. She denied speech changes or dysphagia. She recalled history of encephalitis at age three. She was in special education classes and was unable to complete high school. She had anxiety onset age 19. Examination revealed normal speech, right > left hand dystonia, right foot eversion while ambulating, 4/5 weakness in finger extensors and finger intrinsic muscles on the right, and 2 beats of clonus in the right ankle (Additional files 4, 5, 6). MRI brain showed T2 hyperintensity without enhancement in the deep and subcortical white matter of the left frontal lobe suggesting remote infarct in the left middle cerebral territory (Fig. 3).

**Additional file 4.** Patient’s mother (II-2) has normal speech without dysarthria.



**Additional file 5.** Patient’s mother (II-2) displays right > > left hand dystonia.



**Additional file 6.** Patient’s mother (II-2) displays mild bilateral arm dystonia and right foot eversion while ambulating.


Maternal male cousin, age 4(III-7), had delayed milestones, attention deficit hyperactivity disorder, anxiety, and behavioral concerns. There was history of frequent choking; however, video-fluroscopy was normal. Height was consistently less than the 10th percentile and weight less than the 3rd percentile. Examination revealed hypernasal speech and preferential toe walking, although he was able to walk heel toe when prompted.

Maternal female cousin, age 6(III-6), initially presented for evaluation of hypernasal speech. A submucous cleft palate was identified and she underwent a Furlow palatoplasty without improvement. She had difficulties swallowing as an infant, requiring feed thickener. Dysphagia resolved over time. Growth was consistently below the 10th percentile for height and weight. Early toe walking improved with therapy. She has learning delays and receives therapies and support in school. Vision abnormalities include left-sided strabismic amblyopia, hyperopia, accommodative esotropia, and astigmatism. Neurological evaluation revealed hypernasal, but fluent speech, normal resting tone with normal deep tendon reflexes, and gait with external rotation of the left leg with weight distribution on the lateral aspect of the foot, suggesting mild dystonia.

Signs and symptoms in maternal grandfather and maternal uncles are reported by other relatives; none have been examined by a neurologist. Maternal grandfather I-1 had mild short stature and was aesthenic. Maternal uncle, age 41(II-3), recently lost use of his left arm; he has not had testing for the familial variant. Maternal uncle, age 40(II-4), an obligate carrier of the variant, is intellectually impaired and has short stature. He reported tingling and numbness in the neck that progressed to painful paresthesias in all four limbs onset age 20.

## Methods

### Sequencing

WGS was performed as previously described [8, 9]. Variants were prioritized by allele frequency, conservation, and predicted effect on protein function and confirmed by Sanger sequencing. Phenotypic terms included the following: dysarthria, gait disturbance, failure to thrive, and dysphagia. Given MRI findings, the sequence was re-queried to specifically exclude other variants in genes associated with brain iron accumulation. Subsequent family studies were performed by targeted Sanger sequencing.

### Literature review

We reviewed 80 cases obtained by PubMed Search using term KMT2B as well as cases in the reference list for these manuscripts or otherwise known to the author, that were not identified by PubMed search. (Additional Table).

## Discussion and conclusions

Our report describes the phenotype in a family with a previously undescribed *KMT2B* variant and highlights failure to thrive, an overlooked manifestation. Thus far, 80 additional patients have been described (Additional Table) [5, 6, 10–28]. A recent report identified *KMT2B* mutations in 21.5% of patients with previously undiagnosed childhood-onset dystonia suggesting *KMT2B* mutations may be a relatively common cause of dystonia in children [26]. The natural course of *KMT2B* dystonia involves focal onset lower limb dystonia with progression to generalization. Of reported cases, 23 noted dysphagia, 6 required gastrostomy tube but failure to thrive was rarely mentioned (Additional Table) [5, 6, 10–28]. In our series, the proband and two cousins had dysphagia. Poor weight gain despite normal swallowing study in one cousin and refractory weight gain after gastrostomy tube placement in the proband, suggest factors other than mechanical impairments to swallowing may underlie FTT in this condition.

Progressive dystonia with prominent oromandibular involvement, mild cognitive dysfunction and imaging findings in the proband are consistent with features previously described in *KMT2B* mutation carriers [6]. Other previously described neurologic features noted in the proband include dysfluency, bulbar dysfunction, dysphagia, intellectual disability, and developmental delay [5, 6].^,^ Additional reported features not present include eye movement abnormalities, skin changes, psychiatric co-morbidities (anxiety, depression, attention deficit hyperactivity disorder, obsessive-compulsive disorder), myoclonus, seizures, spasticity, sensorineural hearing loss, microcephaly, and parkinsonism [5, 6, 10–28].

Interestingly, the proband’s mother did not manifest poor weight gain and reported no motor symptoms until age 29. Similarly, maternal uncles report only adult-onset neurologic symptoms. Reduced penetrance, variable expressivity, and adult onset up to 43 years of age have been reported [14, 26]. As neither maternal grandfather, nor either maternal uncle was examined, we cannot confirm whether dystonia is present. Similarly, it is unclear if mother’s signs and symptoms are due to genetic dystonia, unrecognized cerebrovascular accident, or a combination of both given imaging evidence for remote infarct. Vascular insults have not been reported in *KMT2B* mutation carriers though most reported cases are children without long-term follow-up. Further study will be necessary to determine whether *KMT2B* mutation is a risk factor for stroke.

Since molecular diagnosis, the proband has trialed levodopa/carbidopa and trihexyphenidyl without benefit. Deep brain stimulation of globus pallidus (DBS) is reported to improve dystonia in select patients, suggesting another possible avenue for efficacious treatment for affected members of this family [5, 6, 26].

Our case series underscores the importance of careful history and thorough examination when determining etiologies for failure to thrive. In the presence of an abnormal neurologic exam or history of developmental delays, clinicians should strongly consider genetic testing. Unbiased genetic testing in this setting, including whole exome and genome sequencing, has enabled identification of rare disorders, especially those presenting with non-typical phenotypes. This case series highlights the non-neurologic aspects of *KMT2B* mutation and demonstrates the advantages of molecular genetic testing for defining the precise and potentially treatable etiologies of FTT. It also reinforces the importance of cascade screening of family members to bring clarity of unrecognized diagnoses more broadly beyond the presenting family member.

## Supplementary information

**Additional file 7: ** Phenotype and Genotype of Previously Reported *KMT2B* Mutation Carriers and Patients Described in This Report. F-Female; M-Male.

## Data Availability

*KMT2B* variant data has been deposited in ClinVar with accession codes (https://www.ncbi.nlm.nih.gov/clinvar/variation/692033/ Variation ID:692033). Additional data generated or analyzed during this study are available from the corresponding author on reasonable request.

## Abbreviations

*KMT2B*: Lysine Methyltransferase-2B
FTT: Failure to thrive
MRI: Magnetic resonance imaging
WGS: Whole genome sequencing
